# Real-World Evidence of Disproportionate Financial Toxicity and Health-Related Social Needs Among Adolescents and Young Adults Receiving Care at an NCI-Designated Cancer Center

**DOI:** 10.3390/curroncol33080477

**Published:** 2026-08-13

**Authors:** Brittany C. Kimball, Minji K. Lee, Kimberly O. Steinert, Jordan A. Mason, Wendy A. Hanson, Wendy A. Allen-Rhoades, Allison C. Rosenthal, Nandita Khera, Robert R. McWilliams

**Affiliations:** 1 Department of Oncology, Mayo Clinic, Rochester, MN 55905, USA; 2 Department of Quantitative Health Sciences, Mayo Clinic, Rochester, MN 55905, USA; 3 Ascension Health, St. Louis, MO 63134, USA; 4 Comprehensive Cancer Center, Mayo Clinic, Rochester, MN 55905, USA; 5 Pediatric Hematology/Oncology Division, Mayo Clinic, Rochester, MN 55905, USA; 6 Division of Hematology/Oncology, Mayo Clinic, Phoenix, AZ 85054, USA; 7 Department of Medical Oncology, OSF Cancer Institute, Peoria, IL 61637, USA

**Keywords:** adolescent and young adult, financial toxicity, financial strain, medical debt, health-related social needs, food insecurity, transportation needs

## Abstract

Financial toxicity is a significant but often overlooked consequence of cancer for adolescents and young adults (AYAs, 15–39 years old), who face limited savings, unstable employment, and insurance gaps that heighten financial stress. As part of a larger initiative to determine whether our cancer center can use an existing social drivers of health (SDOH) screening process to identify patients who may benefit from targeted supportive interventions, we sought to characterize financial strain, toxicity, and SDOH needs in AYAs treated in our cancer center and compare them to both younger and older cancer patients. This is a cross-sectional observational study at Mayo Clinic of patients with an oncology-related department visit between 8 September 2023–4 June 2024 (*N* = 81,731) who completed routine institutional electronic screenings for financial strain and SDOH. Survey items assessed financial strain, transportation barriers, and food insecurity; institutional billing data captured financial toxicity (unresolved balances, collection referrals, write-offs). AYAs (*n* = 5655) were more likely than non-AYAs to report medium/high financial strain, have unpaid debt, or have an unresolved balance, referral to a collection agency, or bad debt write-off, with nearly double the odds of medium/high financial strain compared to patients over 40 (OR 2.02, 95% CI 1.84–2.23, *p* < 0.001). Within AYAs, ages 26–32 bore the greatest burden: 24.2% in the medium/high-risk category, 9.4% with unresolved billing issues or collections, and 11.6% reporting food insecurity. These findings identify AYAs—particularly those 26–32—as a distinct high-risk group for financial toxicity and health-related social needs, underscoring the need for targeted interventions, tailored financial navigation, and policy efforts to reduce economic barriers to care. Future research should evaluate the effectiveness of these interventions in mitigating financial toxicity, addressing health-related social needs, and improving patient outcomes in this population.

## Simple Summary

Adolescents and young adults (AYAs, ages 15–39) with cancer face distinct financial challenges—limited savings, unstable jobs, and insurance gaps—that increase their risk of financial distress and unmet health-related social needs. Using routine electronic screening and billing data from 81,731 patients (5655 AYAs), this study found AYAs were significantly more likely than older or younger patients to report medium/high financial strain, carry unpaid medical debt, and need pre-visit financial review due to unpaid bills; those aged 26–32 showed the highest burden of financial risk, unresolved balances, collection referrals or write-offs, and food insecurity. These findings identify AYAs as a high-risk group for financial toxicity and health-related social needs, supporting the development of targeted interventions, policy changes to reduce economic barriers to care, and future research on tailored support programs for adolescent and young adult cancer patients.

## 1. Introduction

While recognition of financial toxicity as a consequence of cancer care has grown in the literature, our ability to effectively address it in day-to-day oncology care remains inadequate. Financial toxicity refers to the problems a patient experiences related to the cost of medical care—including debt, bankruptcy, and inability to pay other bills or save money, as well as distress and poorer quality of life [1]. People with cancer experience higher rates of financial toxicity than people without cancer [2,3,4]. Financial toxicity leads to poor medication adherence, care avoidance, social isolation, and psychological distress; and there are even associations between severe financial toxicity and mortality [5,6,7]. It also intersects with other health-related social needs (HRSN), defined by the Centers for Medicare and Medicaid Services (CMS) as including housing instability, food insecurity, utility help needs, and transportation problems [8]. These individual-level needs are shaped by broader social drivers of health, which encompass the systemic conditions in which people live—neighborhood access to things like food, education, and healthcare. Consequently, addressing financial toxicity in cancer care does not rely only on dealing with costs of care and medications, but also addressing food security, transportation, and other HRSN within the complex contexts of people’s lives.

Certain patient populations are disproportionately vulnerable to financial toxicity, with adolescents and young adults (AYAs) representing a particularly high-risk group. AYAs, defined as people ages 15–39 years old at cancer diagnosis, account for 4.2% of cancer diagnoses, with rates of early-onset cancer rising [5,6,9]. Contributing factors to financial burden in this demographic include educational interruptions, lack of savings, work disruption, family responsibilities, insurance limitations, and high out-of-pocket costs [10,11]. Leading cancer organizations recommend screening and targeted interventions to address financial toxicity in AYAs. The Children’s Oncology Group and NCCN Guidelines for AYA Oncology both include recommendations for socioeconomic evaluations and referrals to services such as social work, vocational counseling, and financial support [12,13]. Specific interventions must ultimately be developed for local contexts, but some interventions that have shown positive benefits for improving financial toxicity include financial counseling, financial assistance, and treatment and insurance decision aids [14].

As part of a broader initiative to improve patient outcomes, our institution implemented an electronic distress screening tool administered alongside SDOH/HRSN screening. Within the Mayo Clinic Comprehensive Cancer Center, we are analyzing these data to identify patients at risk for financial toxicity to intervene early. Our overarching goal is to determine whether a cancer center can use an existing SDOH screening process to identify patients who may benefit from targeted supportive interventions. This analysis explores financial risk and HRSN in AYAs treated in our cancer center, compares them to both younger and older cancer patients, and examines risk factors associated with high financial distress or unmet social needs in AYAs.

While financial toxicity and HRSN have been independently described in oncology populations, few studies have leveraged routinely collected, EHR-integrated screening data to examine their intersection, particularly among adolescents and young adults (AYAs). Moreover, direct comparisons between AYA and non-AYA patients within the same clinical setting with tools obtained as part of routine care remain limited. Historical data show that AYAs have not experienced the same survival improvements as either children or older adults [15]. By benchmarking AYAs against both flanking age groups, we can better identify aspects of AYA cancer care that contribute to these inferior outcomes. By using financial distress and SDOH screening embedded in clinical care institution-wide, we designed a pragmatic observational cross-sectional study from existing clinical and financial data to identify whether upfront SDOH questions were associated with downstream financial adverse outcomes as collected in our revenue system. As we know financial strain and impacts can vary by age group, we chose to focus on AYAs in comparison to other groups. We also discuss next steps for addressing these needs in this age group.

## 2. Materials and Methods

### 2.1. Data Collection

As part of usual care, patients received a financial distress screen and an SDOH questionnaire distributed once yearly at any ambulatory visit with options of completing it via the electronic patient portal or in person. Mayo Clinic began screening for SDOH in November 2019. The questionnaire included items assessing financial strain, transportation needs, and food insecurity. For patients under the age of 13, parents serve as proxies. Starting at age 13, only the child has access to the questionnaires, unless they are completed at a visit via a tablet or nursing interview, in which case parents are often providing the information. Data, as it existed in the active medical record on that date, was extracted on 4 June 2024. The most recently completed questionnaire is what exists in the medical record, and thus that is what was used. Patients were included if they were listed in the cancer patient registry and had completed a visit with an oncology-related department between 8 September 2023 and 4 June 2024 at any Mayo Clinic site (*N* = 81,731). Demographic, financial, and diagnosis data were extracted from the medical record, with diagnoses identified using ICD10 codes. Neighborhood-level socioeconomic disadvantage was assessed using the area deprivation index (ADI), assigned based on the patient’s zip code and expressed as national percentile ranks (higher percentiles indicate greater deprivation). Rural–urban commuting area codes (RUCA), updated by the USDA Economic Research Service and derived from Census Bureau data, were also assigned by zip code to classify residential areas as metropolitan, micropolitan, small town, or rural.

### 2.2. Data Sources and Measures

To assess financial strain, patients were asked the recommended CMS and the National Academy of Medicine (NAM) screener question, “how hard is it for you to pay for the very basics like food, housing, medical care, and heating”? Responses were recorded on a 5-point Likert scale ranging from “not hard at all” to “very hard”. Responses of “hard” or “very hard” were categorized as high risk, “somewhat hard” as medium risk, and “not very hard” or “not hard at all” as low risk [16]. This item serves as a proxy for financial strain. Based on recommendations from CMS and NAM, this question has been widely adopted and studied as a financial strain screening tool in a variety of clinical and research settings [17]. A systematic review identified 13 studies using this single-item tool and demonstrated predictive validity, with financial strain linked to poorer mental health, physical health, and cognitive and functional health outcomes [17]. Additionally, pre-appointment review (PAR) flags assigned by the institution when there is an unresolved balance, a referral to a collection agency, or a bad debt write-off—along with total debt amounts—were extracted from billing records and used as indicators of financial toxicity, as these are objective measures of financial outcomes. HRSN were evaluated with questions from the CMS Accountable Health Communities (AHC) HRSN Screening Tool. Transportation was assessed with the questions “In the past 12 months, has lack of transportation kept you from medical appointments or from getting medications?” and “In the past 12 months, has lack of transportation kept you from meetings, work, or getting things needed for daily living?” These questions had response options of “Yes”, “No”, or “Decline”. Food security was assessed with responses to the statements “Within the past 12 months, you worried that your food would run out before you got money to buy more” and “Within the past 12 months, the food you bought just didn’t last and you didn’t have money to get more”. Response options included “Never true”, “Sometimes true”, “Often true”, and “Decline” [16]. This two-question screening tool has been validated in multiple studies [18,19,20].

### 2.3. Analytic Approach

We conducted both descriptive and inferential analyses. For comparisons across age groups, we defined AYA patients as ages from 15 to 39 years old and compared them to non-AYA patients (patients younger than 15, patients 40 years or older). For the primary comparison, we grouped all non-AYA patients (<15 and ≥40) into a single “non-AYA” category to provide a clear, interpretable estimate of the difference between AYAs and the aggregate risk profile of patients outside the AYA range. To address potential heterogeneity between <15 and ≥40 populations, we also performed additional age-stratified analyses comparing AYAs separately to each flanking group (<15 and ≥40). For additional analyses of AYAs, we further stratified patients into four subgroups: adolescents (15–19 years), emerging adults (20–25 years), ‘younger’ young adults (26–32 years), and ‘older’ young adults (33–39 years). These age groups were derived from Siembida et al. but modified so that the ‘younger’ young adult group began at age 26, the age at which young adults can no longer use parental health insurance in the United States [20].

Group comparisons were conducted on demographic and clinical characteristics, financial strain (single item CMS/NAM screener), downstream financial impacts as measured through available revenue cycle database information (PAR flags, which when assigned result in a pause of scheduling of further appointments pending business office clearance; and unpaid bills written off as bad debt), which we termed as financial toxicity for the purposes of this study, and HRSNs (food security and transportation access), using Pearson’s chi-squared test for categorical variables, trend tests for ordinal variables, or Kruskal–Wallis tests for continuous variables. To identify demographic associations with financial risk, unmet transportation needs, food insecurity, active bad debt, and presence of a PAR flag, we conducted multivariable logistic regression analyses with multiple imputation by chained equations (MICE) applied to address missing data, assuming data missing at random. Thirty imputed datasets were generated for each regression analysis using the fully conditional specification algorithm. The imputation models included all variables used in the respective multivariable analyses, including age group, sex, race, primary language, insurance type, ADI (per 10-percentile increase), RUCA classification, and interaction terms between RUCA and ADI to assess effect modification by rurality, as well as the corresponding outcome variable. Imputation methods were automatically assigned by the “mice” package in R according to variable type [21]. Standard MICE diagnostic plots were examined, including density plots and strip plots for the continuous ADI variable and trace plots of parameter means and standard deviations across imputations. Trace plots showed relatively stable means and standard deviations across imputations. Logistic regression models were fitted separately within each imputed dataset, and pooled odds ratios, confidence intervals, and *p*-values were obtained using Rubin’s rules.

## 3. Results

Data for 5655 AYA patients and 76,076 non-AYA patients (965 younger than 15 years old and 75,111 40 years or older) were analyzed at the timepoint described in the Section 2. Demographics are outlined in Table 1. Demographic and clinical differences were noted between the AYA (15–39 years) and non-AYA (<15 years and 40+ years) groups. More AYAs were female—60.3%—than non-AYAs—45.4% (<15 years and 50.0% 40+ years). AYAs were more racially diverse—14.8% non-white AYAs vs. 7.4% non-white 40+ years—and more likely to speak a primary language other than English—4.0% AYAs vs. 2.0% 40+ years—than those over the age of 40, but less so than those younger than 15—14.8% non-white AYAs vs. 18.3% non-white < 15 years and 4.0% non-English primary language AYAs vs. 8.2% in those <15 years. AYAs were the most likely group to carry commercial insurance—79.9% of AYAs compared to 36.1% of those 40+ years and 60.7% of those <15 years. There were also cancer diagnosis differences between the groups. Some patients had multiple cancers based on ICD10 diagnosis codes, and we included all separate diagnoses in demographics. In those younger than 15, the most common cancer types were brain (34.4%), sarcoma (23.0%), leukemia (19.0%), neuroendocrine/adrenal/other endocrine (11.2%), and genitourinary (7.3%). For AYAs, the most common cancer types were brain (17.7%), lymphoma (15.8%), breast (14.4%), sarcoma (14.1%), and gastrointestinal (10.4%). For adults over 40, the most common cancer types were genitourinary (24.1%), breast (18.7%), gastrointestinal (15.8%), lymphoma (13.1%), and lung (8.5%).

### 3.1. AYA vs. Non-AYA Financial Strain and HRSN

AYAs reported a higher proportion of medium and high financial strain compared to both younger and older patients. Overall, the rate of medium and high financial strain was 8.1% and 2.4%, respectively, in the entire cohort. AYAs were more likely than non-AYAs to report a medium (14.7% vs. 7.6%, *p* < 0.001) or high (5.0% vs. 2.2%, *p* < 0.001) financial strain (Table 2). AYAs were also more likely to have unpaid debt (9.2% vs. 4.2%, *p* < 0.001) or a PAR flag (7.9% vs. 3.5%, *p* < 0.001). As shown in Table 3, patients under age 15 years had 39% higher odds of reporting medium or high financial risk compared to those over 40 (OR 1.39, 95% CI 1.08–1.79, *p* = 0.01). However, AYAs faced even greater financial vulnerability, with nearly double the odds of reporting medium/high financial risk relative to patients over 40 (OR 2.02, 95% CI 1.84–2.23, *p* < 0.001).

Food insecurity was present in 3.6% overall, with rates of 8.9% in the AYA group and 3.2% in the non-AYA group, *p* < 0.001. Unmet transportation needs were 2.0% overall, with rates of 3.6% in the AYA group and 1.9% in the non-AYA group, *p* < 0.001. See Table 2 for details. As Table 3 shows, compared to individuals over 40, AYAs had 2.15 times higher odds of experiencing unmet transportation needs (*p* < 0.001, CI 1.80–2.57). Younger individuals, especially those aged 15–39, were at greater risk of food insecurity (OR = 2.82 for AYAs and OR = 1.91 for age < 15). They also had greater risk of having active bad debt (OR = 1.48 for AYAs and OR = 1.55 for age < 15) or PAR flags (OR = 1.48 for AYAs and OR = 1.34 for age < 15). Overall, AYAs faced more financial and transportation challenges, with food insecurity being particularly higher risk, as outlined in Table 3.

Across all models (Table 3), the national percentile rank of the ADI was associated with financial vulnerability outcomes. Each 10-percentile increase in ADI was associated with 1.27 times higher odds of medium/high financial strain, 1.20 times higher odds of unmet transportation needs, 1.34 times higher odds of food insecurity, 1.12 times higher odds of having active bad debt, and 1.19 times higher odds of a PAR flag status. In RUCA-defined rural areas, each 10-percentile increase in ADI is associated with approximately 15% higher odds of medium/high financial strain (OR = 1.27 × 0.91 = 1.15). In small towns, the increase in odds was 19.5% (OR = 1.27 × 0.95 = 1.20), while in metropolitan areas, the increase was 27% (OR = 1.27) per 10-percentile increase in ADI. Non-white race and female gender were associated with higher financial risk, financial toxicity, and unmet HRSN. A non-English primary language was associated with higher financial risk and unmet HRSN. Having government insurance was associated with unmet transportation needs and food insecurity but lower rates of PAR flags and bad debt.

### 3.2. AYA Developmental Age Subgroups and Demographic Associations with Financial Strain and HRSN

Within our AYA cohort (Table 4), 638 (11.28%) were adolescents ages 15–19, 966 (17.08%) were emerging adults ages 20–25, 1485 (26.26%) were ‘younger’ young adults ages 26–32, and 2566 (45.38%) were ‘older’ young adults ages 33–39. Along with having the lowest percentage of medium/high financial strain patients compared to the other groups (9.2%, Table 4), adolescents aged 15–19 years old were also the least likely to have medium/high financial risk compared to the 33–39-year-old patients (OR 0.35, *p* < 0.001, Table 5). Food insecurity was also least likely for this adolescent group (OR 0.53, *p* < 0.05, Table 5). Conversely, the ‘younger’ young adults ages 26–32 had the highest percentage of medium/high financial strain (24.2%, Table 4) and also trended toward higher financial risk, unmet transportation needs, bad debt, and PAR flags, though the statistical comparisons to the 33–39 age group were insignificant (Table 5). However, the likelihood of food insecurity was significantly increased for the 26–32 age group (OR 1.3, *p* < 0.05) compared to the oldest AYA patients.

Within the AYA cohort, non-white race remained an important demographic factor associated with worse financial strain and HRSNs (Table 5). Demographic factors that did not meet statistical significance for being associated with worse outcomes in the AYA cohort compared to the overall cohort included female gender and non-English primary language. AYAs with “other” insurance—largely self pay—had over six times higher rates of unmet transportation needs compared with those with commercial insurance. Also for AYAs, those with government insurance compared to commercial insurance were three times more likely to report medium or high financial risk, and over four times more likely to have unmet transportation needs or food insecurity; they were also less likely to have financial toxicity indicators like bad debt (OR 0.53, *p* < 0.001) or PAR flags (OR 0.66, *p* < 0.05).

## 4. Discussion

### 4.1. Findings

In this analysis from a large NCI-designated cancer center, we found using real-world data that patients under 40—especially AYAs aged 15–39—faced the highest rates of financial strain and toxicity. Within the AYA group, those ages 26–32 reported the highest levels of financial strain and had the highest rates of financial toxicity, as indicated by PAR flags (unresolved balances, accounts in collection, or bad debt write-offs).

Among AYAs, non-white race was associated with having medium/high financial risk, unmet transportation needs, and food insecurity. Government insurance in this AYA cohort was associated with medium/high financial risk, unmet transportation needs, and food insecurity; however, it was also associated with lower financial toxicity in the form of bad debt or PAR flags.

### 4.2. Interpretation

These findings align with prior research showing that financial hardship is a major issue for AYA cancer patients, strengthening the credibility of these findings in a large, real-world sample [12,22]. Compared to older adults, AYAs are more likely to be underinsured, earn less, lack savings, and have limited access to financial buffers like Social Security or retirement income. At the same time, compared to younger children, they are less likely to receive parental financial support [12].

Differences in cancer type and treatment intensity may also contribute to the observed age-related differences in financial outcomes. For example, AYAs may be more likely to receive prolonged, multimodality therapy for malignancies such as sarcoma and acute leukemia, which may increase direct and indirect financial burdens; however, substantial heterogeneity exists both within and across disease types, and disease-specific effects were beyond the scope of this analysis.

AYAs are not a monolithic group, and developmental, insurance, and employment contexts differ within the population’s age range. Like in our study, Kaddas et al. found greater financial burden among older AYAs (26–39) compared to those aged 15–25 [23]. Patients between 26 and 39 years of age are at a stage of life where they may be raising young children, repaying student loans, still in graduate programs, establishing a career, or purchasing homes—just as cancer disrupts everything. They are less likely than younger AYAs to have parental support and more likely to have dependents [24]. Finding and affording childcare for medical appointments is a major issue, particularly with decreased income due to work disruption from cancer. Their parents may be retired, deceased, or even need support themselves. The Affordable Care Act allows young adults to remain on parental insurance until age 26; beyond that, securing coverage becomes more difficult. Thus, it is unsurprising that this transitional period is marked by increased financial burden.

The observed associations between non-white race and higher financial strain and HRSNs likely reflect the effects of broader structural inequities, including differences in socioeconomic opportunity, insurance coverage, access to care, and systemic barriers like neighborhood differences in transportation access. These findings underscore the importance of addressing upstream structural issues that contribute to financial toxicity.

Transportation difficulties were more common among AYAs, which may reflect financial constraints—limited funds for a car, gas, maintenance, and parking—especially as medical costs rise. Some AYAs need rides due to treatment or illness (e.g., brain tumors, chemotherapy) but struggle to find daytime caregivers or drivers. Food insecurity, also higher in AYAs, is associated with an increased likelihood of delaying treatment and higher mortality in cancer survivors [25,26]. This aligns with prior work showing that AYAs report unmet social support needs [12]. Higher social vulnerability has been associated with higher all-cause mortality in AYAs with cancer [27].

### 4.3. Limitations

The pragmatic design of this study is important to understanding real-world experiences of financial hardship and other unmet needs, but it does come with limitations. We utilized pre-existing data within the medical record, which meant we relied on financial distress assessment tools used in routine care. Thus, we did not use a financial toxicity survey tool developed for this research purpose and our financial toxicity measures are administrative indicators of financial toxicity, rather than more multifaceted, patient-reported measures. While these administrative indicators are meaningfully tied to financial hardship in the clinical context, they do not fully capture all pertinent dimensions of financial toxicity, such as out-of-pocket cost burden, lost income, or behavioral consequences like delaying or foregoing care. Additionally, there are other factors not measured here that may also be associated with financial strain, including employment status and family income. Area-level socioeconomic measures such as ADI and RUCA may not fully capture individual-level socioeconomic circumstances.

We also could not engineer the response timing to correspond with important timepoints of care, such as diagnosis, treatment timepoints, or survivorship timepoints. While institution-wide annual screening timepoints pragmatically make sense to avoid survey fatigue, we know that for cancer patients in particular, financial needs change rapidly across the care continuum, from diagnosis through survivorship. There are also cancer-specific financial trajectories. As such, there is a need for repeated longitudinal financial screening of patients with cancer in particular, which was not a part of this study.

Further, this is a cross-sectional, observational study, and thus, while it highlights the issue of financial strain in this population and demonstrates notable associations, we cannot prove causation with this methodology. As we used real-world data for this study, missing data also was a challenge. To address missing data, we relied on multiple imputation, assuming data were missing at random. However, it is possible that missing data was not entirely random. For example, there may be biases or barriers that affect response rates of specific demographic groups, such as those who are seen only once for a consultative visit, people with language barriers, people with disabilities affecting usability of surveys, or those with higher clinical acuity [28,29].

Another limitation is generalizability to other health systems and settings. This study was conducted with data from a single health system, though notably this health system includes tertiary care sites in Minnesota, Arizona, and Florida as well as community sites in Minnesota and Wisconsin; all sites are considered part of the overarching NCI-designated cancer center. Studies have found that costs of care are higher in academic settings [30,31]. Patients requiring complex, and often high-cost, surgical interventions may be referred from community to tertiary centers and this may drive higher costs in settings such as this. However, other studies have noted that actual out-of-pocket costs may be similar between settings or only modestly higher in comprehensive cancer centers [32,33]. Due to the costs of care and travel, there is some basis to expect higher financial strain and for financial toxicity to be magnified in a tertiary care setting. At the same time, there are many patients who do not have the financial or social resources to travel to a tertiary care setting, and thus, in that sense, levels of financial strain may be higher in some community settings. Regardless, while the numbers may look different, we anticipate the trends between AYA and non-AYA patients to be similar across settings. Importantly, we feel that this screening—and subsequent targeted interventions—could be implemented in both community and academic settings. While the resources to address financial stress may be different between settings, identifying those at highest risk and directing inherently limited resources towards those who need them can be applied across the board.

Though we lacked comprehensive instruments and pre-set time points, this pragmatic design enabled analysis of a broad real-world population and a large sample size and incorporated additional pragmatic indicators of downstream measurable financial impacts, which we termed toxicity, like PAR flags and unpaid debt. Further, there is referral bias in this academic center sample, where rare or complex cases requiring multidisciplinary care are overrepresented. In our sample, brain tumors, sarcoma, lymphoma, myeloma, and neuroendocrine cancers were disproportionately represented. Nationally, the most common cancers in AYAs are breast, thyroid, testis, and melanoma [5]. In spite of this referral bias, which may limit generalizability to all care environments, it reflects the reality of complex care environments and remains valid for AYAs treated in tertiary care settings.

### 4.4. Implications

This study highlights the need for more effectively mitigating financial toxicity and addressing HRSN in AYAs. These findings add to a body of evidence highlighting the urgent need to provide tailored, comprehensive support for AYAs with cancer. As early-onset cancers rise, targeted interventions to reduce financial toxicity are urgently needed.

Addressing this complex challenge will require multi-level approaches, including macro-level policy shifts and interventions at individual cancer centers. Our discussion of financial toxicity would be incomplete without addressing policies that impact our patients. At a policy level, AYAs with cancer need stronger protection for sick leave, job security, access to affordable childcare, and access to high-quality, affordable health insurance. Notably, AYAs with government insurance were at higher risk for financial strain and HRSN but also demonstrated less financial toxicity with lower rates of bad debt and PAR flags. Safety net government insurance like Medicaid supports low-income AYAs in maintaining health insurance. Medicaid represents the vast majority of government insurance for AYAs, and primarily low-income people are eligible. Thus, it is not surprising that this low-income group of AYAs was at higher risk for financial strain; having a lower income makes it harder to pay for basics like food, housing, medical care, and heating. However, low-income AYAs who have free or affordable government insurance like Medicaid can pay their medical bills because of these programs, which helps to avoid bad debt in the healthcare setting. Threats to Medicaid—like work requirements or funding cuts—risk worsening financial toxicity. Programs like SNAP provide critical support for AYAs with cancer who are struggling to meet basic needs. Expansion of support, including considering special provisions for AYA cancer survivors, is a foundational element to supporting these vulnerable patients [34]. Other policies that could positively impact the financial health of AYA patients with cancer include mandated coverage for fertility preservation and infertility treatment.

We relied on institutionally collected data, which is implemented for all patients, not just patients treated within the cancer center. We wanted to see if we could use what the institution was already collecting to determine whether it correlated with outcomes using administrative and billing data and, subsequently, to identify those patients at highest risk so that we could target limited resources to those who need it most. Financial professionals and clinicians can work together to design convergent systems that meet the needs of patients. In an “aha” moment, one of our financial collaborators voiced, “Perhaps we should use the PAR flag to connect patients with our support resources instead of restricting appointment scheduling”. Cancer centers can integrate routine financial distress screening into multidisciplinary support pathways so that limited resources can be routed towards the patients who need it most. As a result of the findings in this manuscript, our institution is developing cancer center-wide interventions using the available clinical information to generate automatic referrals to financial counselors, patient navigation, or social work based on screening responses. We are also building AYA-focused resources.

These findings present an opportunity for cancer centers to pilot targeted interventions for AYAs. Potential strategies include scheduling automatic visits with financial navigators trained to address the complex realities AYAs face—such as student loan deferment, insurance transitions, and unpaid bills. Similarly, automatic visits with social workers may help ensure AYAs are aware of federal, state, and community resources such as medical rides and SNAP benefits. Prospective multicenter intervention trials are needed for evaluating whether early financial navigation and other supportive measures can improve both patient-reported outcomes and treatment adherence. Complementary efforts might involve directing patients to age- or disease-specific financial resources, deploying in-depth financial risk screening tools, and implementing longitudinal follow-up assessments to monitor ongoing risk. This is also an opportunity to explore additional funding for material support for vulnerable AYAs, such as transportation vouchers, waived parking fees, grocery gift cards, or childcare for appointments. Additionally, proactive counseling for 25-year-olds nearing the end of parental insurance coverage could help mitigate abrupt transitions and prevent financial toxicity. Finally, partnering with programs that support young people continuing school or work during or after treatment could offer tailored guidance, resume support, and accommodation recommendations to make that transition more attainable and sustainable.

Fortunately, most AYA patients go on to become long-term cancer survivors. Better outcomes are linked to fewer care interruptions, stronger treatment adherence, and robust survivorship care, all of which require mitigating financial toxicity throughout differing stages of care and survivorship. We must take proactive measures from the outset to prevent financial hardship in a healthcare system not designed for this population navigating young adulthood.

## 5. Conclusions

In this cross-sectional, observational study at an NCI-designated cancer center, AYAs—especially those aged 26–32—were found to face disproportionate financial strain, financial toxicity, and HRSN compared to other age groups. Given that our analyses were performed within a single health system and show associations rather than causal mechanisms, future studies should include multiple institutions with more robust inclusion of non-academic centers and examine the pathways driving age-related differences to inform targeted, age-appropriate intervention design. These results underscore the need for prospective, multicenter intervention trials designed to measure the effect of financial navigation and support for HRSN on patient-reported financial outcomes and treatment adherence in AYAs with cancer.

## Figures and Tables

**Table 1:** Demographic characteristics (<15 years, 15–39 years, 40+ years).

	Age < 15 (*n* = 965)	Age 15–39 (*n* = 5655)	Age 40+ (*n* = 75,111)	Total (*n* = 81,731)	*p*-Value
**Sex**					<0.001 ^1^
Female	438 (45.4%)	3411 (60.3%)	37,578 (50.0%)	41,427 (50.7%)	
Male	527 (54.6%)	2239 (39.6%)	37,530 (50.0%)	40,296 (49.3%)	
Nonbinary	0 (0.0%)	3 (0.1%)	0 (0.0%)	3 (0.0%)	
Unknown/Missing	0 (NA)	2 (NA)	3 (NA)	5 (NA)	
**Race**					<0.001 ^1^
African/African American/Black	72 (7.8%)	360 (6.6%)	2633 (3.6%)	3065 (3.9%)	
American Indian	11 (1.2%)	71 (1.3%)	456 (0.6%)	538 (0.7%)	
Asian	58 (6.3%)	252 (4.6%)	1694 (2.3%)	2004 (2.5%)	
Other	25 (2.7%)	105 (1.9%)	503 (0.7%)	633 (0.8%)	
Pacific Islander	2 (0.2%)	14 (0.3%)	85 (0.1%)	101 (0.1%)	
White	751 (81.7%)	4624 (85.2%)	67,576 (92.6%)	72,951 (92.0%)	
Unknown/Missing	46 (NA)	229 (NA)	2164 (NA)	2439 (NA)	
**Ethnicity**					<0.001 ^1^
Hispanic or Latino	116 (12.6%)	564 (10.4%)	2944 (4.1%)	3624 (4.6%)	
Not Hispanic or Latino	803 (87.4%)	4853 (89.6%)	69,442 (95.9%)	75,098 (95.4%)	
Unknown/Missing	46 (NA)	238 (NA)	2725 (NA)	3009 (NA)	
**Primary Language**					<0.001 ^1^
English	866 (91.8%)	5318 (96.0%)	72,551 (98.0%)	78,735 (97.8%)	
Non-English	77 (8.2%)	223 (4.0%)	1463 (2.0%)	1763 (2.2%)	
Unknown/Missing	22 (NA)	114 (NA)	1097 (NA)	1233 (NA)	
**RUCA Category**					<0.001 ^1^
Metropolitan	582 (62.6%)	4028 (73.1%)	52,686 (70.9%)	57,296 (71.0%)	
Micropolitan	157 (16.9%)	637 (11.6%)	9591 (12.9%)	10,385 (12.9%)	
Small Town	96 (10.3%)	468 (8.5%)	6402 (8.6%)	6966 (8.6%)	
Rural Area	95 (10.2%)	376 (6.8%)	5628 (7.6%)	6099 (7.6%)	
Unknown/Missing	35 (NA)	146 (NA)	804 (NA)	985 (NA)	
**ADI National Rank (Unscaled)**					<0.001 ^2^
Unknown/Missing	635 (NA)	998 (NA)	6390 (NA)	8023 (NA)	
Mean (SD)	54.591 (20.959)	48.112 (22.624)	44.644 (23.628)	44.907 (23.579)	
Median	54	47	44	44	
Q1, Q3	41.000, 70.000	31.000, 65.000	26.000, 62.000	26.000, 63.000	
Range	2.000–100.000	1.000–100.000	1.000–100.000	1.000–100.000	
**Current Coverage Primary Financial Class (Grouped)**					<0.001 ^1^
Commercial… (Commercial and Mayo Employee)	529 (60.7%)	4268 (79.9%)	26,466 (36.1%)	31,263 (39.3%)	
Government… (Medicaid, Medicare, and Medicare Advantage)	320 (36.7%)	943 (17.7%)	46,179 (62.9%)	47,442 (59.6%)	
Other… (Self-Pay, Embassy, and Misc. Government)	22 (2.5%)	129 (2.4%)	760 (1.0%)	911 (1.1%)	
Unknown/Missing	94 (NA)	315 (NA)	1706 (NA)	2115 (NA)	
**Cancer type—number (% of known)**					<0.001 ^1^
Brain/nervous system	467 (17.7%)	114 (34.4%)	1120 (2.0%)	1701 (2.4%)	
Breast	379 (14.4%)	0 (0.0%)	10,607 (18.7%)	10,986 (15.5%)	
Gastrointestinal	275 (10.4%)	6 (1.8%)	8983 (15.8%)	9264 (13.1%)	
Genitourinary	165 (6.3%)	24 (7.3%)	13,726 (24.1%)	13,915 (19.7%)	
Gynecologic	107 (4.1%)	1 (0.3%)	2912 (5.1%)	3020 (4.3%)	
Head & neck	88 (3.3%)	0 (0.0%)	2963 (5.2%)	3051 (4.3%)	
Leukemia	227 (8.6%)	63 (19.0%)	1578 (2.8%)	1868 (2.6%)	
Lung	26 (1.0%)	1 (0.3%)	4812 (8.5%)	4839 (6.8%)	
Lymphoma	416 (15.8%)	9 (2.7%)	7429 (13.1%)	7854 (11.1%)	
Sarcoma	371 (14.1%)	76 (23.0%)	2303 (4.1%)	2750 (3.9%)	
Skin	83 (3.1%)	4 (1.2%)	4237 (7.5%)	4324 (6.1%)	
Thyroid	61 (2.3%)	0 (0.0%)	924 (1.6%)	985 (1.4%)	
Myeloma/dysproteinemia	45 (1.7%)	1 (0.3%)	3665 (6.5%)	3711 (5.2%)	
Neuroendocrine/other endocrine	89 (3.4%)	37 (11.2%)	1360 (2.4%)	1486 (2.1%)	
Other	70 (2.7%)	14 (4.2%)	866 (1.5%)	950 (1.3%)	
Unknown	3017 (NA)	634 (NA)	18,256 (NA)	21,907 (NA)	

**Notes:** Statistical Tests ^1^ Pearson’s chi-squared test. ^2^ Kruskal–Wallis rank sum test; *p*-values were computed excluding missing/unknown responses from the calculations.

**Table 2:** AYA vs. non-AYA financial strain, financial toxicity, food insecurity, and transportation needs.

	AYA (15–39) (*n* = 5655)	Non-AYA (<15 or 40+) (*n* = 76,076)	Total (*N* = 81,731)	*p*-Value
**Financial Strain**				<0.001 ^1^
Low Risk	3141 (80.3%)	49,210 (90.2%)	52,351 (89.6%)	
Medium Risk	576 (14.7%)	4142 (7.6%)	4718 (8.1%)	
High Risk	196 (5.0%)	1183 (2.2%)	1379 (2.4%)	
Unknown/Missing/Declined	1742 (NA)	21,541 (NA)	23,283 (NA)	
**PAR Flag**				<0.001 ^1^
No	5208 (92.1%)	73,409 (96.5%)	78,617 (96.2%)	
Yes	447 (7.9%)	2667 (3.5%)	3114 (3.8%)	
**Active Bad Debt**				<0.001 ^2^
None or $0	5136 (90.8%)	72,873 (95.8%)	78,009 (95.4%)	
$0.01–$99.99	68 (1.2%)	485 (0.6%)	553 (0.7%)	
$100–$499.99	93 (1.6%)	692 (0.9%)	785 (1.0%)	
$500–$999.99	70 (1.2%)	450 (0.6%)	520 (0.6%)	
$1000–$4999.99	198 (3.5%)	1032 (1.4%)	1230 (1.5%)	
>$5000	90 (1.6%)	544 (0.7%)	634 (0.8%)	
**Food Insecurity**				<0.001 ^1^
Present	407 (8.9%)	1952 (3.2%)	2359 (3.6%)	
Absent	4151 (91.1%)	58,317 (96.8%)	62,468 (96.4%)	
Unknown/Missing	1097 (NA)	15,807 (NA)	16,904 (NA)	
**Transportation Needs**				<0.001 ^1^
No Needs	4547 (96.4%)	60,597 (98.1%)	65,144 (98.0%)	
Unmet Needs	169 (3.6%)	1162 (1.9%)	1331 (2.0%)	
Missing/Unknown	939 (NA)	14,317 (NA)	15,256 (NA)	

**Notes:** Statistical Tests ^1^ Pearson’s chi-squared test. ^2^ Trend test for ordinal variables; *p*-values were computed excluding missing/unknown responses from the calculations.

**Table 3:** Logistic regression analyses for demographic factors associated with medium/high financial strain, unmet transportation needs, and food insecurity in the entire cohort.

	Medium/High Financial Risk (OR)	Unmet Transportation Needs (OR)	Food Insecurity (OR)	Active Bad Debt (OR)	PAR Flag (OR)
**Age 15–39 (Ref: 40+)**	2.022 **	2.150 **	2.821 **	1.482 **	1.475 **
**Age < 15 (Ref: 40+)**	1.390 *	0.946	1.905 **	1.551 **	1.336 *
**Race: Non-White (Ref: White)**	1.759 **	2.325 **	2.773 **	1.649 **	1.562 **
**Primary Language: Non-English (Ref: English)**	1.704 **	1.960 **	1.655 **	1.005	1.048
**Female (Ref: Male)**	1.408 **	1.707 **	1.589 **	1.104 *	1.096 *
**Insurance: Government (Ref: Commercial)**	1.048	2.279 **	1.544 **	0.387 **	0.352 **
**Insurance: Other (Ref: Commercial)**	0.86	2.723 **	0.908	0.767	0.543 *
**RUCA: Micropolitan (Ref: Metropolitan)**	0.905	0.755	0.949	0.994	1.447
**RUCA: Small Town (Ref: Metropolitan)**	1.195	0.611	0.851	1.083	1.817 *
**RUCA: Rural Areas (Ref: Metropolitan)**	1.415	1.235	1.199	1.522	2.092 *
**ADI National Rank, per 10 percentile increase**	1.265 **	1.203 **	1.340 **	1.123 **	1.193 **
**Interaction: Micropolitan × ADI**	0.979	1.015	0.970	0.976	0.937 *
**Interaction: Small town × ADI**	0.945 *	1.032	0.990	0.953	0.912 *
**Interaction: Rural areas × ADI**	0.909 *	0.950	0.915	0.908	0.896 *

* *p* < 0.05, ** *p* < 0.001; Ref: reference category used in the regression model; ADI (area deprivation index) is scaled in 10-percentile units; odds ratios reflect the effect of each 10-point increase for ADI, where a higher percentile rank indicates greater socioeconomic disadvantage.

**Table 4:** AYA developmental age groups financial strain, financial toxicity, food insecurity, and transportation needs.

	Adolescents (15–19) (*n* = 638)	Emerging Adults (20–25) (*n* = 966)	Younger Young Adults (26–32) (*n* = 1485)	Older Young Adults (33–39) (*n* = 2566)	Total AYA Only (15–39) (*n* = 5655)	*p*-Value
**Financial Strain**						<0.001 ^1^
**Low Risk**	376 (90.8%)	531 (82.5%)	795 (75.8%)	1439 (79.7%)	3141 (80.3%)	
**Medium Risk**	29 (7.0%)	88 (13.7%)	187 (17.8%)	272 (15.1%)	576 (14.7%)	
**High Risk**	9 (2.2%)	25 (3.9%)	67 (6.4%)	95 (5.3%)	196 (5.0%)	
**Unknown/Declined/** **Missing**	224 (NA)	322 (NA)	436 (NA)	760 (NA)	1742 (NA)	
**PAR Flag**						<0.001 ^1^
**No**	611 (95.8%)	905 (93.7%)	1346 (90.6%)	2346 (91.4%)	5208 (92.1%)	
**Yes**	27 (4.2%)	61 (6.3%)	139 (9.4%)	220 (8.6%)	447 (7.9%)	
**Active Bad Debt**						0.002 ^2^
**None or $0**	603 (94.5%)	887 (91.8%)	1325 (89.2%)	2321 (90.5%)	5136 (90.8%)	
**$0.01–$99.99**	8 (1.3%)	7 (0.7%)	22 (1.5%)	31 (1.2%)	68 (1.2%)	
**$100–$499.99**	5 (0.8%)	15 (1.6%)	26 (1.8%)	47 (1.8%)	93 (1.6%)	
**$500–$999.99**	4 (0.6%)	14 (1.4%)	24 (1.6%)	28 (1.1%)	70 (1.2%)	
**$1000–$4999.99**	10 (1.6%)	35 (3.6%)	65 (4.4%)	88 (3.4%)	198 (3.5%)	
**>$5000**	8 (1.3%)	8 (0.8%)	23 (1.5%)	51 (2.0%)	90 (1.6%)	
**Food Insecurity**						<0.001 ^1^
**Food Insecurity Present**	29 (5.7%)	62 (8.0%)	141 (11.6%)	175 (8.5%)	407 (8.9%)	
**No Food Insecurity**	484 (94.3%)	717 (92.0%)	1072 (88.4%)	1878 (91.5%)	4151 (91.1%)	
**Unknown/Declined/** **Missing**	125 (NA)	187 (NA)	272 (NA)	513 (NA)	1097 (NA)	
**Transportation Needs**						0.056 ^1^
**No Transportation Needs**	530 (98.0%)	781 (95.4%)	1192 (96.0%)	2044 (96.7%)	4547 (96.4%)	
**Unmet Transportation Needs**	11 (2.0%)	38 (4.6%)	50 (4.0%)	70 (3.3%)	169 (3.6%)	
**Unknown/Declined/** **Missing**	97 (NA)	147 (NA)	243 (NA)	452 (NA)	939 (NA)	

**Notes:** Statistical Tests ^1^ Pearson’s chi-squared test. ^2^ Trend test for ordinal variables; *p*-values were computed excluding missing, unknown, or patient-declined responses from the calculations, aside from the PAR flag and active bad debt variables, where no flag or no balance reported was considered no flag or 0, respectively.

**Table 5:** Multivariable logistic regression analyses for demographic factors associated with medium/high financial strain, unmet transportation needs, and food insecurity among AYA individuals (ages 15–39 years).

	Medium/High Financial Risk (OR)	Unmet Transportation Needs (OR)	Food Insecurity (OR)	Bad Debt (OR)	PAR Flag (OR)
Adolescents Age 15–19 (Ref: 33–39)	0.351 **	0.496	0.533 *	0.587 *	0.47 **
Emerging Adults Age 20–25 (Ref: 33–39)	0.853	1.371	0.947	0.852	0.717 *
‘Younger’ Young Adults Age 26–32 (Ref: 33–39)	1.206	1.087	1.287 *	1.141	1.09
Race: Non-White (Ref: White)	1.33 *	2.649 **	1.883 **	1.227	1.173
Primary Language: Non-English (Ref: English)	0.619	1.29	0.933	0.503 *	0.938
Female (Ref: Male)	1.168	0.999	1.225	1.079	1.035
Insurance: Government (Ref: Commercial)	3.037 **	4.201 **	4.106 **	0.526 **	0.664 *
Insurance: Other (Ref: Commercial)	1.262	6.144 **	1.905 *	0.781	0.301 *
RUCA: Micropolitan (Ref: Metropolitan)	0.914	0.285	0.843	1.518	2.874
RUCA: Small Town (Ref: Metropolitan)	0.797	0.034	0.221	1.421	1.438
RUCA: Rural Areas (Ref: Metropolitan)	1.072	0.836	0.44	0.708	1.548
ADI National Rank, per 10 percentile increase	1.185 **	1.171 *	1.207 **	1.074 *	1.11 **
Interaction: Micropolitan × ADI	0.929	1.145	0.979	0.914	0.83 *
Interaction: Small town × ADI	0.956	1.465	1.14	0.889	0.948
Interaction: Rural areas × ADI	0.922	0.98	1.042	1.021	0.919

* *p* < 0.05, ** *p* < 0.001; Ref: reference category used in the regression model; ADI (area deprivation index) is scaled in 10-percentile units; odds ratios reflect the effect of each 10-point increase for ADI, where a higher percentile rank indicates greater socioeconomic disadvantage.

## Data Availability

The data presented in this study may be available from the corresponding author upon reasonable request. The data are not publicly available because they contain potentially identifiable patient information, and sharing is restricted to protect participant privacy and confidentiality.

## Abbreviations

The following abbreviations are used in this manuscript:

HRSN	Health-related social needs
CMS	Centers for Medicare and Medicaid Services
AYA	Adolescent and young adult
SDOH	Social drivers of health
ADI	Area deprivation index
RUCA	Rural–urban commuting area codes
NAM	National Academy of Medicine
PAR	Pre-appointment review
AHC	Accountable Health Communities
